# Evolutionary trajectory of transcription factors and selection of targets for metabolic engineering

**DOI:** 10.1098/rstb.2023.0367

**Published:** 2024-11-18

**Authors:** Yun Sun Lee, Edward L. Braun, Erich Grotewold

**Affiliations:** ^1^ Department of Biochemistry and Molecular Biology, Michigan State University, East Lansing, MI 48824, USA; ^2^ Department of Biology, University of Florida, Gainesville, FL 32611, USA

**Keywords:** phylogenetic relationship, biosynthesis pathway, anthocyanins, specialized metabolism

## Abstract

Transcription factors (TFs) provide potentially powerful tools for plant metabolic engineering as they often control multiple genes in a metabolic pathway. However, selecting the best TF for a particular pathway has been challenging, and the selection often relies significantly on phylogenetic relationships. Here, we offer examples where evolutionary relationships have facilitated the selection of the suitable TFs, alongside situations where such relationships are misleading from the perspective of metabolic engineering. We argue that the evolutionary trajectory of a particular TF might be a better indicator than protein sequence homology alone in helping decide the best targets for plant metabolic engineering efforts.

This article is part of the theme issue ‘The evolution of plant metabolism’.

## 1. Introduction

Plant biotechnology is in large part predicated on the manipulation of metabolic pathways, irrespective of whether the final objective is to make plants with increased biomass, plants more resistant to the environment, or simply plants that accumulate larger quantities of a particular compound. While the idea of identifying pathway rate-limiting steps as a primary target of manipulation is attractive and has been successfully applied in many instances for both primary and specialized metabolism (reviewed in [1–4]), flux control theory distributes responsibility across most, if not all, pathway enzymes [5]. According to the flux summation theorem, increasing the efficiency of one pathway step immediately creates bottlenecks elsewhere. A logical solution to the constraints imposed by the theory is to increase the quantity or efficacy of all pathway steps simultaneously [6], something that can theoretically be accomplished by manipulating the regulator(s) of all genes encoding pathway enzymes. Transcription factors (TFs) provide promising candidates as pathway regulators and have been proposed as powerful alternatives to targeting single enzymes for metabolic engineering [7,8]. However, our ability to predict which TF is idoneous for a particular metabolic engineering effort is still deficient, and much of the success with using TFs to manipulate pathways has been fortuitous rather than predictable [8]. The subject of this essay is to evaluate to what extent, and when, phylogenetic relationships can assist us in predicting the TF that most likely will deliver the desired outcomes in plant metabolic engineering efforts.

## 2. Transcription factors are part of large families

TFs are proteins that bind DNA in a sequence-specific fashion. Most TFs reside in the nucleus, although increasingly it is being recognized that a significant fraction of TFs dwell in different cellular locations, and translocate to the nucleus upon particular stimuli [9]. About 7% of all the genes in a vascular plant encode for TFs which can be classified into 50–60 families based on amino acid sequence similarities in their DNA-binding domains and/or in protein–protein interaction regions necessary for regulatory function [10,11]. Protein–DNA interactions often involve an α-helix that inserts into the major groove of the DNA and makes contact with 3–5 nucleotides, although other DNA-recognition elements, some capable of recognizing the minor grove, are also known [12]. Often, two or more DNA-interaction surfaces participate in DNA binding, either as part of the same polypeptide (e.g. the two Myeloblastosis [MYB] repeats in R2R3-MYB TFs) or because the TF binds DNA as homo- or hetero-dimer (e.g. basic Helix-Loop-Helix [bHLH] domain in bHLH TFs). *In vitro*, TFs recognize short (6-12 bp) DNA-sequence motifs, and members of the same TF family often display very similar, if not identical DNA-binding specificities [13,14]. Thus, predicting the genes that a TF will regulate just from the analysis of DNA-sequence motifs is a widely practised, yet risky, endeavour. TFs control gene expression as part of large regulatory complexes, and while the number of regulatory proteins that simultaneously are recruited to a gene regulatory region is likely to vary between genes, it can involve tens, if not hundreds of proteins [15,16]. Often, however, one or a few of those TFs are the ones that impact gene expression most dramatically, when for example mutated. We will refer to those here as the focal TFs.

## 3. Evolution of transcription factor families

A significantly larger fraction of plant genes encode TFs than animals, with plant TF families expanding faster [17,18]. Many TF families, such as Heat Shock Factor (HSF), bHLH, basic Leucine Zipper (bZIP), Homeobox, and MCM1, AGAMOUS, DEFICIENS, and SERUM RESPONSE FACTOR (MADS), are present in plants, animals, and most or all other eukaryotic lineages. Since the root of the eukaryotic tree is likely to lie between plants and animals [19], the origin of those TF families must predate the earliest divergences among eukaryotes. However, some plant TFs, like MYB TFs, have diverged significantly from their common ancestors with animals. The defining feature of MYB proteins is the presence of one or more MYB repeats, each formed by three α-helices, in which the second and third helix adopts a helix-turn-helix structure with the third α-helix making DNA contacts when they bind DNA [20,21]. Canonical MYB TFs have two or three MYB repeats (R1, R2, and R3). Vertebrates express three MYB TFs (A-MYB, B-MYB, and c-MYB), and are therefore called R1R2R3-MYB, 3R-MYB, or MYB3R [22,23]. Plants express a small number (approx. five) of 3R-MYB TFs that, like animals, have been implicated in the regulation of the cell cycle [22,24]. However, the vast majority of the plant MYB TFs belong to the R2R3-MYB type [23,25,26], which probably arose from the loss of R1 in the ancestor of the green lineage followed by a few distinctive changes in the MYB domain [27]. Indeed, the Arabidopsis regulators of stomatal patterning FOUR LIPS (FLP) and MYB88 constitute likely intermediates in this evolutionary process as they have R2R3-MYB domains, but share intron/exon structure and MYB-domain protein features with 3R-MYB TFs, and they also regulate very particular aspects of the plant cell cycle [28].

Certain TFs (e.g. TEOSINTE BRANCHED1, CYCLOIDEA, and PROLIFERATING CELL FACTOR 1/2 [TCP], NO APICAL MERISTEM, ARABIDOPSIS TRANSCRIPTION ACTIVATION FACTOR 1/2, CUP-SHAPED COTYLEDON [NAC], LEAFY [LFY], AUXIN RESPONSE FACTOR [ARF], GIBBERELLIN-INSENSITIVE, REPRESSOR OF GA INSENSITIVE, and SCARECROW [GRAS], and WRKY) are unique to the plants, and possibly involved in the regulation of plant-specific processes [18,29–31]. Some plant TF families expanded in early land plant lineages (e.g. WRKY, MADS) [32], approximately 400 million years ago (Mya), while others, such as plant-specific DNA binding with One Finger (DOF) TFs, expanded later with a vascular system development [33]. Expansion of TF gene families can occur by several mechanisms, including whole genome duplications (WGD), tandem duplications, duplications mediated by transposons, and retroduplication [18]. Ancient WGDs, often associated with polyploidy, played a key role in plant diversification and metabolic diversity [34,35]. This is nicely exemplified by the evolution of insect resistance in the Brassicales provided by glucosinolates (GSLs) [36]. Indole and aliphatic GSLs are independently controlled by partially redundant groups of R2R3-MYB TFs which form complexes with a particular group of bHLH factors that interact (by mechanisms distinct from anthocyanin regulators, see the following text) indistinctively with indole and aliphatic R2R3-MYB regulators (reviewed in [37]). Highlighting the impact of TF evolution on metabolic diversity, the R2R3-MYB TFs associated with GSL accumulation in the Camelineae tribe are significantly less conserved than among the rest of the Brassicales and the loss of MYB34 explained the absence of indole GSLs in members of this tribe [38]. Another example of multiple mechanisms participating in TF expansion is provided by the P-to-A clade of R2R3-MYB TFs, distinguished by a proline (P) to alanine (A) residue in the hinge region joining the R2 and R3 MYB repeats [39], and which expanded during the radiation of the grasses, within the past 35 Mya [40,41].

**Figure 1.**
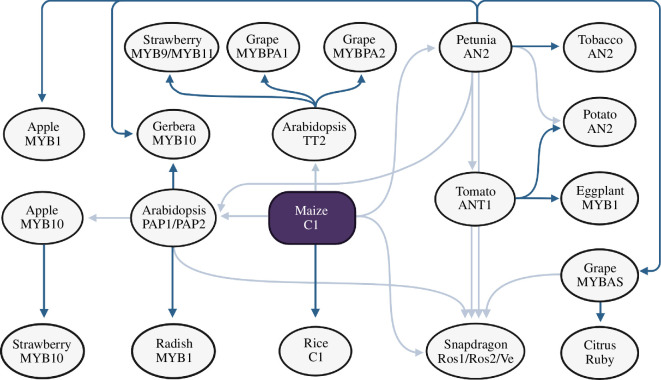
Identification of maize C1 homologous genes and their use for metabolic engineering in various plant species. R2R3-MYB TFs corresponding to maize C1 were identified by sequence similarity-based gene predictions followed by functional characterization (dark blue arrows) or by mutant/population-based gene identification followed by phylogenetic relationship analysis (light blue arrows). The references for the TFs mentioned are as follows: C1 from maize [46] and rice [47]; AN2 from tobacco [48], petunia [49] and potato [50]; ANT1 from tomato [51]; MYB1 and MYB10 from apple [52–54]; PAP1/PAP2 [55] and TT2 [56] from Arabidopsis; MYBAs [57,58] and MYBPAs [59,60] from grape; Rubys from citrus [61]; MYB10 from gerbera [62]; MYB1 from eggplant [63]; MYBs from strawberry [64,65]; MYB1 from radish [66] and Ros1/Ros2/Ve from snapdragon [67].

During evolution, duplication can give rise to genes with different fates [42]. The most common is gene loss, but often the function of the original gene is partitioned between the new copies, most commonly by acquiring complementary expression patterns in a process known as subfunctionalization. Most rare is neofunctionalization, or the process by which a duplicate acquires a new function. There are several examples of recently duplicated plant TF genes that apparently have acquired novel regulatory functions, represented by MADS/SRF [43], APETALA2/Ethylene Responsive Factor (AP2/ERF) [44], and R2R3-MYB TFs [45]. In particular, high functional divergence appears to be evident for many TF families involved in the control of specialized metabolism in angiosperms [18]. Inferring the evolutionary lineages of these TFs and classifying them into multiple sub-groups can be attempted as a preliminary way to identify novel orthologous and paralogous TFs and has thus been extensively exploited (figure 1). They provide good examples of how understanding the evolutionary trajectory can assist in making the correct TF selection for metabolic engineering purposes.

## 4. Homology helps predict function but has limitations

For the highly divergent TFs, phylogenetic reconstructions are usually carried out using the alignable conserved DNA-binding domains, because the DNA-binding domain of a TF corresponding to just a fraction of the protein is highly conserved and the rest of the protein shows limited similarity. A representative example is provided by bHLH TFs, whose sub-groups have been established based on sequence homology of bHLH signature domains, approximately 60 amino acids long [68]. The classification of the bHLH TFs is supported by the finding that TFs belonging to the same sub-group share some degree of functional similarity [26]. However, complex evolutionary mechanisms have acted across or beyond the protein-coding region [69] and may involve few amino acid changes but significant functional changes, which complicate functional predictions based on the phylogenetic inference of the conserved domains (discussed in the following sections).

More specifically, combinatorial gene control of transcriptional regulation often involves interactions of TFs with many other proteins, including other TFs, for efficient and specific gene regulation [70]. Many of these protein–protein interactions are mediated by motifs outside of the conserved DNA-binding domain. Examples include some members of the R2R3-MYB TFs in grapes and apples, which belong to the group consisting of anthocyanin-related TFs (sub-clade 6). However, these TFs failed to activate anthocyanin pigmentation because of amino acid variations in the C-terminal region [71], further highlighting the risks of solely relying on phylogeny for functional prediction.

There are many examples where one or more amino acid changes in the DNA-binding domain with minimal effects on alignment have dramatic effects on DNA-recognition preference [72–75]. Instances like this include the maize TFs P1 and C1 [76]; beet (*Beta vulgaris*) MYB1 [77]; peach (*Prunus persica*) MYB10.1 and MYB10.2 [78]; and the periwinkle (*Catharanthus roseus*) octadecanoid derivative-responsive *Catharanthus* AP2-domain (ORCAs) of the AP2/ERF family [79]. The amino acids in the exemplary MYB TFs are important for the interaction with bHLH TFs, which are R2R3-MYB partners, consequently affecting the specificity [76–78]. In the case of ORCAs, the transactivation activity is affected [79]. A few examples in which phylogeny can misinform functions are described in more detail in the following sections.

### (a) Anthocyanin and betalain regulation by R2R3-MYB and bHLH factors

Because of their conspicuous red-to-purple colour and because they are dispensable under many growth conditions, anthocyanin pigments have been the favourites of plant geneticists [80] and floriculturists [81]. Not surprisingly, anthocyanins also led the way regarding the use of TFs for metabolic engineering [82]. Anthocyanin accumulation is controlled in all plant species studied so far by the combinatorial action of R2R3-MYB and bHLH TFs [83], and WD40 proteins, resulting in what has been called the MBW complex [23,84]. These regulatory genes controlling flavonoid biosynthesis were initially identified in maize (R2R3-MYB TF, *C1/Pl* [46,85]; bHLH TF, *R/B* [86,87]) and further snapdragon (*Antirrhinum majus*), petunia (*Petunia hybrida*) and Arabidopsis by mutant analysis or homology-based searches ([88,89]; figure 1). Most of the R2R3-MYBs that participate in the regulation of anthocyanin biosynthesis belong to sub-group 6 [23] and are characterized by the presence of a set of conserved MYB-domain residues [76,90] that are necessary for the interaction with members of sub-group IIIf of bHLHs [26,84]. Based on these characteristics, it is easy to identify R2R3-MYB and bHLH factors that have the potential to control anthocyanin accumulation, and this has led to the extensive identification of many anthocyanin regulators from many different plant species, including *Brassica* species [91] and Rosaceae family [64].

**Figure 2.**
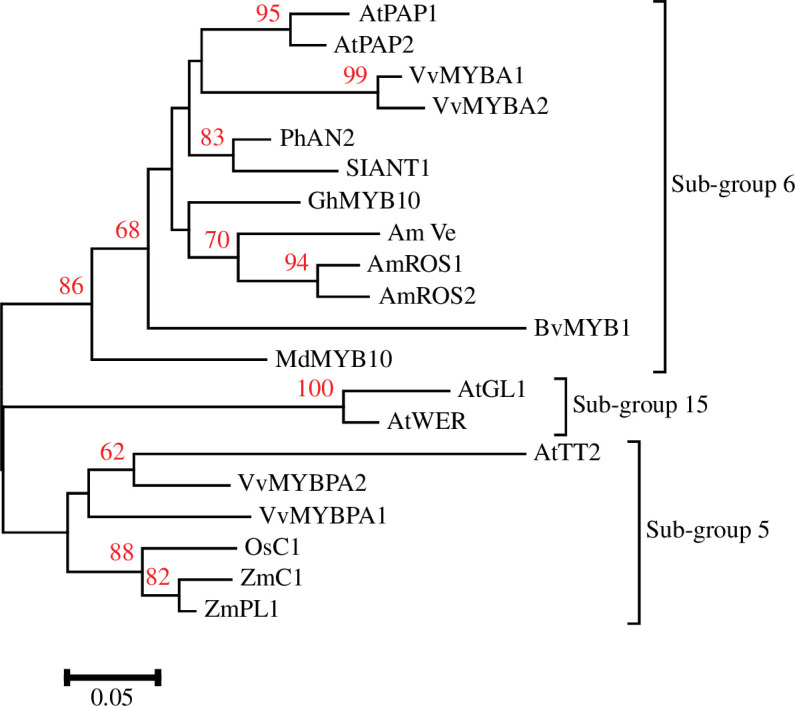
Phylogenetic relationship of flavonoid R2R3-MYBs TFs from different species. The amino acid sequences of the R2R3-MYB domains were aligned using MUSCLE, and the phylogenetic tree was reconstructed using the neighbour-joining method and 1000 bootstraps implemented in MEGA (v. 7.0). Bootstrap values greater than 50 are displayed on the corresponding phylogenetic branch. The species information and accession numbers of the proteins shown are as follows: Arabidopsis: AtTT2 (AT5G35550), AtPAP1 (AT1G56650), AtPAP2 (AT1G66390), AtGL1 (AT3G27920), AtWER (AT5G14750); maize: ZmC1 (GRMZM2G005066), ZmP1 (GRMZM2G084799); beet: BvMYB1 (M1ETK3.1); rice: OsC1 (XP_015642631.1); gerbera: GhMYB10 (CAD87010); snapdragon: AmROS1 (ABB83826.1), AmROS2 (ABB83827.1), AmVe (ABB83828.1); petunia: PhAN2 (AAF66727.1); grape: VvMYBPA1 (CAJ90831.1), VvMYBPA2 (NP_001267953.1), VvMYBA1 (BAE96751.1), VvMYBA2 (BAD18978.1); apple: MdMYB10 (ABB84753.1) and tomato: SlANT1 (WDP81135.1).

Efforts to use phylogeny for gene identification have been reported, and extensive studies suggest complex functional diversification in some clades. The TFs that control anthocyanin biosynthesis provide an excellent example and highlight the need for caution when using phylogeny to predict functions. C1, the first R2R3-MYB TF characterized, forms an MBW complex that regulates maize anthocyanin biosynthesis. Arabidopsis TFs with similar functions include PAP1, PAP2, MYB113, and MYB114 [55,92]. All of these MYBs are somewhat clustered in phylogenies [23,25]. However, C1 is more closely related to TT2, which regulates proanthocyanidin biosynthesis [25,56], and the C1/TT2 clade is distinct from the PAP1 clade (figure 2). C1-activated *BANYULS* (*BAN*), a core proanthocyanidin biosynthesis gene [93], when maize Sn (member of the *R/B* family of bHLH factors) was co-expressed in Arabidopsis, while other Arabidopsis MYB TFs (*PAP1, PAP2, WER, GL1, MYB23,* and *MYB111*) could not activate *BAN* [94]. These results highlight distinct functional specifications of C1 and PAP1, with C1 retaining the ability to regulate proanthocyanidin formation [94], although maize does not accumulate proanthocyanidins [95]. The potential to use phylogeny to predict functions for these TFs without any other information is unclear; the fact that they cluster in a global R2R3-MYB phylogeny might be viewed as sufficient to motivate experiments. A more interesting question is whether the phylogeny is correct. Obviously, the conserved region of R2R3-MYBs is short and that will lead to stochastic errors in phylogenetic estimate, but the potential for artefacts that affect the position of clades does exist. Selection for convergence can result in phylogenetic errors [96] and the possibility that convergence might affect estimates of TF phylogeny should not be discounted.

A prominent example of duplicated TFs involved in lineage-specific metabolite accumulation is observed for betalain, a tyrosine-derived pigment that accumulates specifically in the Caryophyllales [97]. Betalain-producing species do not accumulate anthocyanins because of the loss and degeneration of key anthocyanin enzymatic genes and associated TFs [98–100]. It is noteworthy that BvMYB1 in beets is a member of the same phylogenetic clade as Arabidopsis PAP1 (figure 2; [77]) and lacks the ability to interact with bHLH partners owing to a few amino acid mutations [77] and the loss of GL3 orthologues, the possible bHLH partners [98]. This evolutionary complexity hypothesis illustrates the case where the neofunctionalization of a TF cannot be captured by simple phylogenetic inference and shows how sequence conservation can mislead in target selection for metabolic engineering.

### (b) Terpenoid indole alkaloids by APETALA2/Ethylene-Responsive Factor (AP2/ERF) transcription factors

The AP2/ERF superfamily, one of the largest TF families, is characterized by a conserved AP2/ERF DNA-binding domain, typically comprising approximately 70 amino acids [101]. In plants, the AP2/ERF proteins are categorized into multiple groups based on the conservation of this domain, the exon/intron structure of the respective genes, and protein motif configuration [102]. Notably, clade 2 of group IXa encompasses TFs that play a role in jasmonate-induced plant defence [103], and studies identified several jasmonate-responsive ERF TFs in periwinkle responsible for regulating terpenoid indole alkaloid (TIA) production. Similarly, their homologues in different species (e.g. tobacco, tomato, potato, and *Artemisia annua*) are implicated in the regulation of different alkaloid classes, such as steroidal glycoalkaloids in tomato and potato, nicotine in tobacco, and artemisinin in *A. annua* [103,104].

In periwinkle, *ORCA3* forms a gene cluster in the same genome scaffold as the four ORCAs mentioned above [105]. ORCA3, ORCA4, and ORCA5 cluster in the phylogenetic tree [106,107], all exhibit transcriptional activity and are implicated in the regulation of TIA biosynthesis [105,106]. Recent studies have shown that they share roles in the regulation of the early pathway genes but have distinct functions for late pathway genes [79,106], with the difference also manifested in their specific target gene activation [79,106,107], spatial expression patterns [79] and varying responses to methyl-jasmonate (MeJA) and ethylene [105–107]. The paralogous ERFs in tobacco also show differences in the transcriptional activity and the metabolite induction patterns in response to MeJA [44]. This demonstrates that the functional redundancy and unique functions of individual TFs are not necessarily captured by phylogenetic relationships [106,107].

The phenomenon of AP2/ERF gene clustering is not unique to periwinkle (or to AP2/ERF TFs), and it is observed in several of the aforementioned plant species [44,103,105,108]. Despite their different phylogenetic relationships, the ERFs from different plant lineages have overlapping yet distinct regulatory functions. The overlapping functions are evidenced by the cross-species modulation of gene expression owing to the shared DNA-binding specificity among these TFs between species [106]. The distinct properties are demonstrated by their tissue-specific expression, which correlates with the biosynthesis sites of their specific alkaloid targets [104], and by the distinct *cis*-regulatory element motifs that they recognize [103]. In addition, a recent study suggests that the functional diversification of ORCA3 may be driven by changes in a few critical amino acids. Specifically, swapping unique amino acids in ORCA3 with those of other ORCAs [79] or ERFs from different clades [103] alters its ability to activate gene transcription, suggesting that minor sequence variations lead to notable changes in function, a trend that may not necessarily reflected in phylogenetic relationships.

## 5. Challenges for the use of transcription factors for predictive metabolic engineering

The picture that emerges from the studies described here and many more that we had no opportunity to discuss, is one in which, when the function of a TF has been fully characterized by loss- or gain-of-function approaches, the evolutionary relationships captured by sequence homology and proximity in phylogenetic reconstructions provide a robust predictor of broad function. However, such broad functional knowledge does not necessarily imply that the TF will yield meaningful results in metabolic engineering efforts. A few reasons include the following.

### (a) Metabolic transcription factors target many genes

An unexpected finding of the last decades is that, even for TFs that phenotypically appear to be involved in controlling just one or a few compounds, genome-wide expression analyses demonstrate that they regulate hundreds, if not thousands of genes (table 1). Some of the genes with altered expression could be indirectly controlled by the TF, for example, because the metabolite directly or indirectly regulates gene expression. An example is provided by the effect of flavonols on auxin transport in Arabidopsis [114,115]. However, in the few instances in which direct targets of these TFs have been examined by techniques such as chromatin immunoprecipitation coupled with sequencing (ChIP-seq) or yeast−1-hybrid assays (each technique with its own limitations [116]), these TFs have been shown to directly control many more genes than just those of the pathway [109–111,117,118]. A consequence of this is that when the TF is being used for metabolic engineering purposes, many pathways/processes besides those intended to be manipulated are affected, resulting in several undesirable effects, not the least by carbon being channelled away to undesired pathways, by the perturbation of developmental genes and by the selection of transformants with low transgene expression. Potential solutions to these limitations are to express the transgene from temporally controlled, tissue-specific promoters ensuring that the secondary effects have minimal impact on plant growth and development.

**Table 1.** Number of targets of TFs involved in specialized metabolite biosynthesis.

name	family	pathway	number of differentially expressed genes	% overlap with ChIP-seq targets	reference
maize P1	R2R3-MYB	phenolic compounds	positively regulated: 741	26	[109]
			negatively regulated: 1320	28	
petunia ODORANT1	R2R3-MYB	floral volatile benzenoid and phenylpropanoid compounds	positively regulated: 634	10	[110]
			negatively regulated: 519	10	
maize BZR1	bHLH	brassinosteroid	positively regulated: 1354	43	[111]
			negatively regulated: 1389	34	
cotton PGF	bHLH	terpenoid	positively regulated: 2308	13	[112]
			negatively regulated: 1168	10	
Arabidopsis HBI1	bHLH	brassinosteroids	positively regulated: 600	26	[113]
			negatively regulated: 657	3	

### (b) Moving transcription factors between species can have unpredictable consequences

Because of all the reasons described throughout this study, expressing a TF from one plant species in another, even when the function of the TF is well known, remains a risky business. Most of the time the focus of the researcher is to look for the effect on the pathway of interest, but when inspecting plants in more detail, significant alterations tend to be obvious. Notable examples are provided by the ectopic expression of maize anthocyanin regulators *R/Lc* and its paralogous *B-Peru*, and *C1*. The ecotypic expression of *R/Lc* induced high levels of pigment formation in maize-cultured cells [82], but failed to induce anthocyanin production in tomato fruits, resulting instead in high flavonol accumulation [119]. While *R/Lc* and *C1* can control the maize flavonol synthase (*FLS1*), this is by no means the main function of these TFs in maize and lines without the anthocyanin regulators can make flavonols [120]. In alfalfa, when *R/Lc*, *B-Peru* and *C1* were overexpressed, anthocyanin and proanthocyanidin were excessively accumulated only in plants transformed with *R/Lc* under cold and highlight conditions, but not with *B-Peru* and *C1* [121]. This suggests that although *R/Lc* and *B-Peru* are paralogous genes that are commonly involved in the accumulation of anthocyanins and differ only in their expression sites in maize [122–124] when expressed in plants of different species, they can lead to entirely different yields.

## 6. Evolutionary trajectories for transcription factors

**Figure 3.**
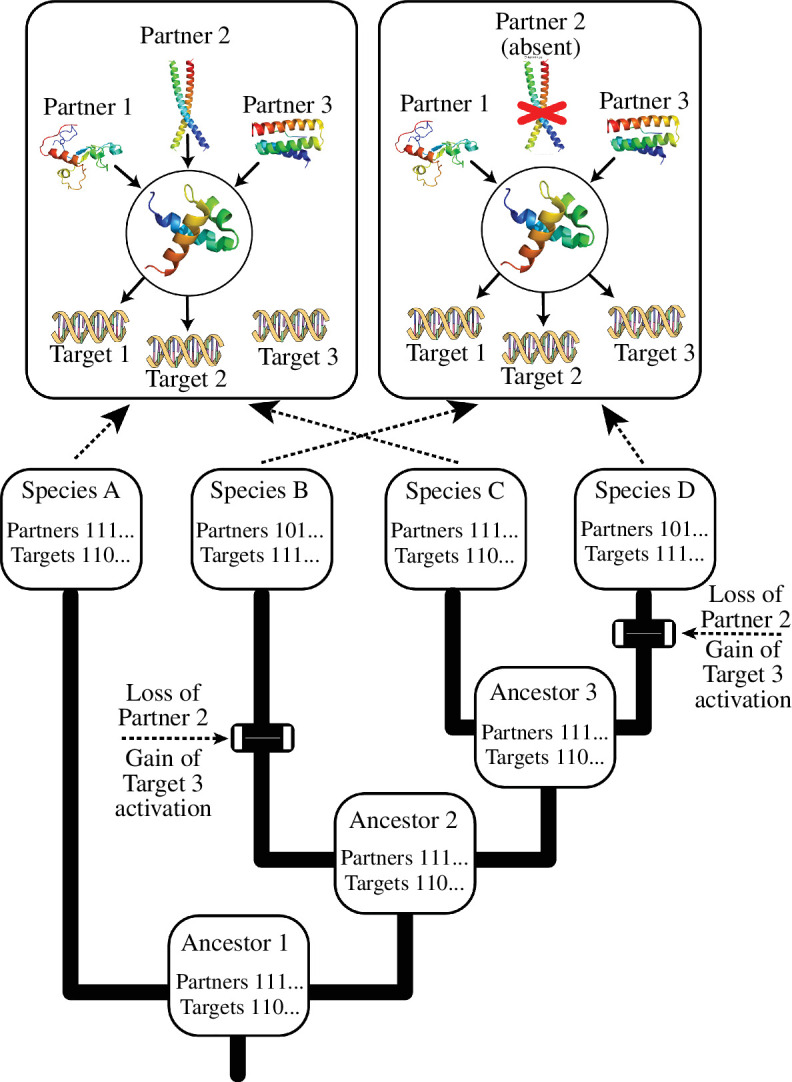
Phylogenetic tree illustrating the evolutionary trajectory of a TF. The focal TF is circled, and it has at least three potential interaction partners and at least three potential target genes. Most TFs would have more partners and targets (the ellipses in the partner and target vectors indicate that additional partners and targets may exist). The focal TF exists in two different states (see arrows) in the four species. This illustrates a hypothetical scenario where partner 2 represses the ability of the focal TF to activate target 3. We also assume that there are no gene duplications, a single gene loss (the loss of partner 2 in species B and D) and that ancestral populations do not remain polymorphic for the presence or absence of partner 2 for long periods of time. Experimental data from the four taxa would allow one to infer that the partner 2 interaction represses target 3 activation; observing similar data with additional taxa would lead to increased confidence in that hypothesis. Other scenarios that might influence target 3 activation are discussed in the text.

The observations that TFs typically target large numbers of genes and often depend on protein–protein interactions suggest a way forward towards models of TF evolution (figure 3). In addition to the sequence of any specific focal TF, the *in vivo* state of that TF can be encoded as two vectors of information, one linking the TF to its targets and a second describing the interactions with its interaction partners. The reason why the transfer of TFs between species can be unpredictable is illustrated by considering the transfer of the focal TF from species B or D to species A. The ability of the B or D TF to activate target 3 depends on whether the B or D TF can bind partner 2. The ancestral version of the focal TF is likely to interact with partner 2, but it is possible that the interaction surface for the focal TF was lost in species B but retained (by chance) in species D; if that is the case target 3 would be activated when the B TF is moved into species A but would not be activated when the D TF is moved into species A.

This complex set of interactions is part of the evolutionary trajectory for TFs, and it limits the predictability of TF function when phylogeny is used in the absence of other information. We intentionally presented a simple example where there are no gene duplications involving the focal TF. However, even this simple example presents challenges for efforts to predict the biological activity of the focal TF. This discussion of evolutionary trajectories illustrates an important point: most of the evolutionary events did not involve the focal TF. Of course, there is every reason to assume that substitutions in the focal TF can alter this complex web of interactions; the issue is the predictability of the phenotypes given the sequence alone.

**Figure 4.**
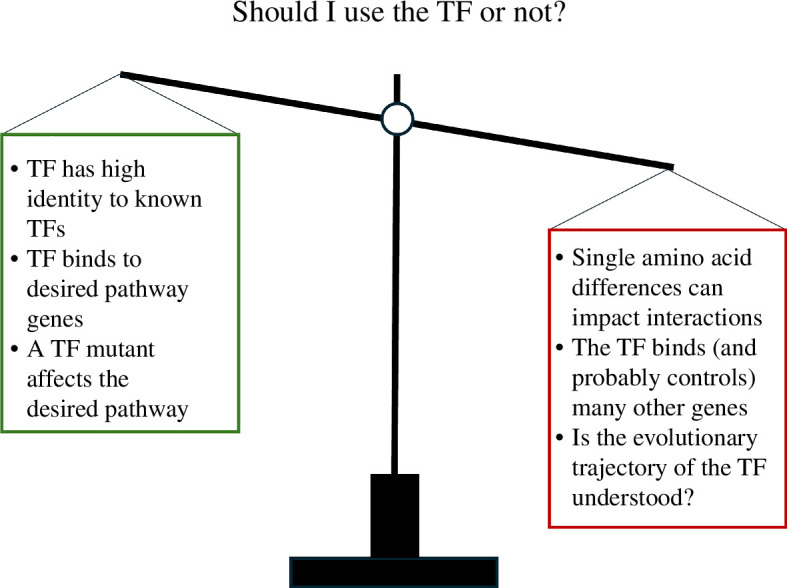
Cartoon illustrating the pros and cons of relying on TF homology and phylogeny to select targets for plant metabolic engineering.

Attempts to model the evolution of this larger network are challenging because all components of the network can change. Although we postulated that activation of target 3 was a consequence of the absence of partner 2, there are many evolutionary changes that might lead to target 3 activation by the focal TF. For example, the promoter of target 3 could change to allow the focal TF to bind and activate target 3 transcription despite the presence of partner 2. Substitutions that affect the interaction surface for the focal TF might lead to the loss of the connection with partner 2. In principle, duplication of the gene encoding partner 2 combined with neofunctionalization might result in a new interaction partner that blocks the connection. In the final scenario, the neofunctionalized partner 2 paralogue would bind to the focal TF and be compatible with target 3 activation; this would lead to a competitive inhibitor of the connection to the fully functional partner 2 paralogue (i.e. the paralog that has retained the ancestral function inhibiting target 3 activation). Our goal in this discussion is not to propose a full analytical approach, it is simply to emphasize that it is possible to devise methods to analyse the evolutionary trajectories of TFs and assist in the selection of TFs as targets for metabolic engineering (figure 4).

## 7. Concluding remarks and open questions

TFs are traditionally classified into families—some families appear to be associated with more essential and/or ancestral functions (e.g. homeodomain TFs), while others appear to be associated more with rapidly evolving traits such as response to environmental responses and specialized metabolism (e.g. R2R3-MYB). However, even within a particular TF family, there is heterogeneity with a good example provided by bHLH TFs—some bHLH TFs are involved in key developmental processes (e.g., epidermal cell fate determination [125]), and others in specialized metabolism (e.g., flavonoids [26]). Should the TF family be considered as a whole, or would not it be more informative to consider the evolutionary trajectory of each individual TF before determining to what extent phylogeny can contribute to predicting metabolic engineering outputs? For example, it might be easier to predict function and therefore phenotypes associated with ectopic expression, of ancient and often conserved TFs while this might be much more challenging for rapidly evolving/less-conserved TFs. As artificial intelligence becomes a more common tool in our toolbox, phylogenetic relationships of TFs must be one of the inputs to be used for training models to predict potential outcomes of using TFs in metabolic engineering. However, this will have to be used with caution as the time and money costs associated with selecting the wrong TF are large (figure 4). Today, trial and error is pretty much the norm, but it is very likely that the risks associated with selecting a TF will have to be much lower in the future.

## Data Availability

This article has no additional data.
